# Assessment of Nonoccupational Exposure to DDT in the Tropics and the North: Relevance of Uptake via Inhalation from Indoor Residual Spraying

**DOI:** 10.1289/ehp.1002542

**Published:** 2010-12-17

**Authors:** Roland Ritter, Martin Scheringer, Matthew MacLeod, Konrad Hungerbühler

**Affiliations:** Safety and Environmental Technology Group, ETH Zurich, Zurich, Switzerland

**Keywords:** biomonitoring, DDT, exposure science, modeling, persistent organic pollutants

## Abstract

**Background:**

People who live in dwellings treated with indoor residual spraying (IRS) of DDT [1,1,1-trichloro-2,2-bis(*p*-chlorophenyl)ethane] for disease–vector control in the tropics and indigenous populations in the Arctic who comsume marine mammals experience high nonoccupational exposure to DDT. Although the use of DDT in IRS is rising, the resulting nonoccupational exposure is poorly characterized.

**Objectives:**

We have provided a comparative assessment of exposure to DDT and its metabolites in the general population of the tropical and northern regions and in highly exposed populations in these regions.

**Methods:**

We compiled > 600 average or median DDT concentrations from the peer-reviewed literature, representing > 23,000 individual measurements in humans, food, air, soil, and dust. We use Monte Carlo sampling of distributions based on these data to estimate distributions of population- and route-specific uptake. We evaluate our exposure estimates by comparing them with biomonitoring data.

**Results:**

DDT concentrations are highest in people living in IRS-treated houses and lowest in the northern general population, differing by a factor of about 60. Inuits and the general population in the tropics have similar concentrations. Inhalation exposure explains most of the difference in concentration between the highly exposed and the general population in the Tropics. Calculated exposure levels are consistent with human biomonitoring data.

**Conclusions:**

Nonoccupational inhalation exposure is a relevant exposure pathway for people living in homes treated by IRS of DDT. Continued monitoring of time trends and DDE to DDT ratios in the Tropics and in the North is needed to identify a possible slowdown in concentration decline and the influence of ongoing DDT use.

The insecticide 1,1,1-trichloro-2,2-bis(*p*-chlorophenyl)ethane (*p,p*′-DDT) was banned in most industrialized countries in the 1970s, mainly because of concerns about its effects on wildlife (Turusov et al. 2002). In 2004, DDT was globally regulated under the Stockholm Convention on persistent organic pollutants (POPs) (United Nations Environment Programme 2010). Under the convention, the only accepted use of DDT is indoor residual spraying (IRS) for disease–vector control. DDT use in IRS in malaria-endemic regions is credited with saving millions of lives (Rogan and Chen 2005; Turusov et al. 2002). In 2006, the World Health Organization (WHO) reaffirmed the importance of DDT use in IRS (WHO 2006). Currently, at least 14 countries use DDT for disease–vector control, and others are preparing to reintroduce it (van den Berg 2009).

DDT is transported over long distances in the environment and has been detected all over the world (Simonich and Hites 1995). As a result, human populations are exposed to DDT and its persistent transformation product, 1,1-dichloro-2,2-bis(*p*-chlorophenyl)ethylene (*p,p*′-DDE), not only in regions where DDT was or is still being used, but also in distant regions.

In the past 15 years, the number of investigations that have focused on DDT and its health effects on humans has increased steadily (Yi and Xi 2008). Kelce et al. (1995) demonstrated that *p,p*′-DDE is a potent androgen receptor antagonist. This finding shifted the focus of research toward the investigation of endocrine-disrupting effects of DDT (Yi and Xi 2008). A growing body of evidence indicates that exposure to DDT and its metabolites may be associated with adverse human health outcomes, particularly in children (Bouwman and Kylin 2009; Eskenazi et al. 2009). This situation requires policy makers and health authorities to balance the risks and benefits of DDT use in malaria control (Rogan and Chen 2005). In addition, because of the long-range transport of DDT in the environment and its accumulation in the marine-food chain, human populations in the Arctic have had considerable exposure to DDT in their diet. This exposure also needs to be evaluated and taken into account in a comprehensive human health assessment of DDT.

As an element of such a comprehensive assessment, we have compared human exposure to DDT in four populations: a population living in houses treated by IRS with DDT, a general population from tropical regions; a population from Greenland that consumes marine mammals in the diet; and a general population from northern regions.

Usually nonoccupational exposure assessments assume that exposure to DDT occurs mainly through diet (Darnerud et al. 2006; Kannan et al. 1997). However, Sereda et al. (2009) recently proposed that for individuals living in IRS-treated houses, other exposure routes, including inhalation or dermal contact, may be important. Therefore, we estimated contributions from three different routes—diet, inhalation, and dermal contact—for the four populations and compared the contributions of the different routes for each population. For each population, we then combined the route-specific uptakes to an estimate of total DDT uptake and, using a pharmacokinetic (PK) model, converted this total uptake into DDT concentrations in the human body. Finally, for all four populations, we evaluated the plausibility of our estimates of DDT concentrations in humans by comparing them with human biomonitoring data. In addition, we analyzed DDE to DDT ratios in the biomonitoring data to distinguish exposure caused by recent or ongoing DDT application from exposure from earlier DDT applications.

## Methods

### Empirical data

We performed an extensive review of the peer-reviewed literature to collect measurements of DDT in exposure media, including food, continental air, indoor air, indoor dust, and soil, and in human tissues. This effort produced a database of > 600 mean or median values representing > 23,000 individual measurements. Measurements in exposure media were collected for the period 1995–2008, and biomonitoring data represent the period 1960–2008. Different analytical chemistry techniques and reporting conventions were used in different studies. Thus, we used ∑DDT to identify the sum of the parent compound DDT and its major breakdown products DDE and DDD [1,1-dichloro-2,2-bis(*p*-chlorophenyl)ethane], and DDTs to nonspecifically refer to this group of substances.

### Geographically integrated exposure assessment

We grouped the empirical data into two geographical regions: the “Tropics” defined as India, Southeast Asia, Africa, South America, and Central America (geographically, South Africa and Swaziland are outside the Tropics but are included here), and the “North,” defined as Greenland, Northern Europe, Canada, and Alaska. Within each region, we further grouped the human biomonitoring data into a general population and a highly exposed population (Table 1). The highly exposed population in the Tropics (THEP) represents individuals living in IRS-treated houses. In the North, the highly exposed population (NHEP) represents Inuits from Greenland who consume marine mammals in their diet [Arctic Monitoring and Assessment Programme (AMAP) 2009]. People living in non–IRS-treated homes in the Tropics, and non-Inuits in the North are taken as representing the general population in the Tropics (TGP) and the North (NGP), respectively. No other subpopulations within the two main regions are resolved by our analysis.

The goals of our geographically integrated exposure assessment are *a*) to identify the dominant DDT exposure routes for the four populations, *b*) to compare the exposure of the four populations, and *c*) to assess for each population the consistency of our exposure estimates against the DDT levels measured by biomonitoring. To do so, we first estimated exposure to DDT through diet, inhalation, and dermal contact for the four populations from our region-specific database of DDT concentrations in exposure media (Table 1). We then used a PK model to calculate population-specific body concentrations of DDT and compared these with the empirical biomonitoring data.

### Construction of total DDT uptake distribution

The DDT concentration data collected from the literature show considerable variability [see Supplemental Material, Table 1 (doi:10.1289/ehp.1002542)]. To estimate total DDT uptake for each population, we first fitted distributions to the DDT concentration data. For most exposure media, these were log-normal distributions (see Supplemental Material, Figure 1). For indoor dust and soil in IRS-treated dwellings, the number of measurements was not sufficient to fit log-normal distributions. Instead, we used a uniform distribution ranging from a minimum to a maximum amount of DDT present on the skin as a result of contact with soil or dust. The distributions of DDT concentrations in exposure media were then multiplied by exposure factors such as inhalation rates and food consumption rates, which yielded distributions of DDT uptake via each individual pathway. For some of the exposure factors, we used point estimates specific to each population, for example, food consumption rates; for others such as the inhalation rate, we used generic average values. Finally, to derive a distribution of total DDT uptake, the individual uptake distributions were combined. The exact solution to this problem would be to calculate the total uptake distribution as the convolution of all individual uptake distributions. We approximated this solution for the total uptake distribution using Monte Carlo sampling, that is, we drew randomly selected values from each individual uptake distribution, calculated the total uptake as their sum, and repeated this 2,000 times.

Because we used point estimates for all exposure factors, variability in DDT concentrations collected from each geographical area is the only source of variability in the distributions of estimated uptake. Thus, our analysis is not a full probabilistic analysis of variability in human exposure to DDT at the individual level, because the entire range of variability in exposure factors within each population is not represented. Our analysis merely converts the empirically observed variability in DDT concentrations in exposure media into a corresponding distribution of uptake of DDT by different routes. This is subsequently translated by the PK model to a distribution of concentrations in the body that is compared with biomonitoring data for DDT in humans. All parameter values used in the derivation of the total uptake distributions for the four populations are given in Supplemental Material, Tables 2, 3, and 4 (doi:10.1289/ehp.1002542); DDT concentrations in exposure media are given in Supplemental Material, Table 1.

### Calculation of route-specific uptake

Uptake is defined as intake multiplied by a factor that represents the bioavailability for the specific route, that is, the uptake efficiency. The daily uptake of ∑DDT from each food group is calculated as


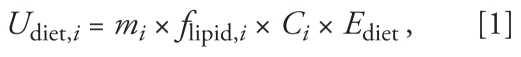


where *U*_diet,_*_i_* (nanograms per person per day) is ∑DDT uptake from food group *i; m_i_* (grams food per person per day) is the average amount of food from food group *i* consumed per day; *f*_lipid_*,_i_* (grams lipid per gram food) is the average lipid fraction of food group *i; C_i_* (nanograms per gram lipid) is the lipid-normalized concentration of ∑DDT in food group *i*; and *E*_diet_ (dimensionless) is the dietary uptake efficiency, estimated at 0.9 (Moser and McLachlan 2001). Food consumption rates, *m_i_*, specific to the tropical and northern populations were derived from the Global Environment Monitoring System(GEMS)/Food Consumption Cluster Diets (WHO 2010) and, for the NHEP, from specific information about diet of Inuit in Greenland [see Supplemental Material, Section 3 (doi:10.1289/ehp.1002542)]. Each cluster represents consumption rates that are based on population averages from a group of countries and rely on food balance sheets of the United Nations Food and Agriculture Organization (WHO 2010). Use of these food consumption rates is in accordance with WHO guidelines for predicting long-term dietary intakes of pesticide residues on the international level (WHO 1997). Values of the food consumption rates are given in Supplemental Material, Table 2.

Uptake via inhalation is estimated from concentration in air according to


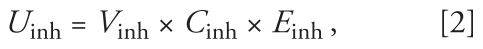


where *U*_inh_ (nanograms per person per day) is ∑DDT uptake via inhalation; *V*_inh_ (cubic meters per person per day) the inhalation rate; *C*_inh_ the appropriate ∑DDT concentration in air; and *E*_inh_ the uptake efficiency. For all populations except the THEP, we assumed indoor and outdoor concentrations of DDT in air are the same. For outdoor air, only gas-phase concentrations of ∑DDT were available. Thus, *C*_inh_ is the concentration in the gas phase in ambient air from the appropriate region, and *E*_inh_ is 1, as recommended by Volckens and Leith (2003) for gas-phase DDT. For the THEP, we used the same assumptions to calculate uptake during the fraction of time spent outdoors. For air inside of IRS-treated homes, concentrations of DDT in the gas and particulate phase have been reported (Singh et al. 1992). We therefore estimated uptake by inhalation of gas and particle-phase DDT for the THEP separately during the time spent indoors (8 hr/day), using *E*_inh_ = 1 for gas phase DDT and *E*_inh_ = 0.44 for particle-phase DDT (Volckens and Leith 2003). These two uptakes are then added to yield the overall inhalation uptake from the time spent indoors.

Dermal uptake of ∑DDT via indoor dust or soil in IRS-treated dwellings is estimated according to Equation 3 (with soil and dust treated as one exposure medium):


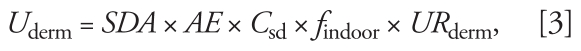


where *U*_derm_ (nanograms per person per day) is the ∑DDT uptake via dermal contact to indoor soil and dust; *SDA* (milligrams per square centimeter) is the soil and dust adherence, that is, the amount of soil and dust present on a square centimeter of skin; *AE* (square centimeters per person) is the area of skin exposed to indoor soil/dust; *C*_sd_ (nanograms per milligram soil) is the concentration of ∑DDT in soil and dust; *UR*_derm_ (1/day) is the experimentally derived rate constant for uptake of DDT from soil by human skin (Spalt et al. 2009). Maximum values of *SDA*, *AE*, and *C*_sd_ define the upper bound of *U*_derm_; minimum values of the three parameters define the lower bound. A uniform distribution of *U*_derm_ between these two bounds is used in the Monte Carlo sampling.

### Calculation of body concentrations

For each of the 2,000 Monte Carlo iterations, the estimated total uptake of ∑DDT was translated into an estimated concentration in the body by means of a steady-state PK model. The model requires dose-weighted intrinsic elimination half-lives (Lorber 2002) to describe elimination of ∑DDT. We used elimination half-lives that range from 4.1 years for the THEP, which is exposed to high proportions of the more rapidly eliminated *p,p*′-DDT, to 6 years for the NHEP, for which *p,p*′-DDE is the major compound ingested. These population-specific half-lives were based on estimates of intrinsic elimination half-lives of *p,p*′-DDT (2.2 years) and *p,p*′-DDE (6.2 years) (Ritter et al. 2009) and on measured ratios of *p,p*′-DDE to *p,p*′-DDT [for details, see Supplemental Material, Section 4 and Tables 5 and 6 (doi:10.1289/ehp.1002542)].

After results from 2,000 Monte Carlo iterations were collected, modeled distributions of body concentrations in the four populations were compared with the range of ∑DDT measurements from the biomonitoring data [see Supplemental Material, Table 7 (doi:10.1289/ehp.1002542)].

We performed the Monte Carlo calculations using Matlab 2010 (Mathworks Inc., Natick, MA, USA).

## Results

### Summary of biomonitoring data

**Figure 1:**
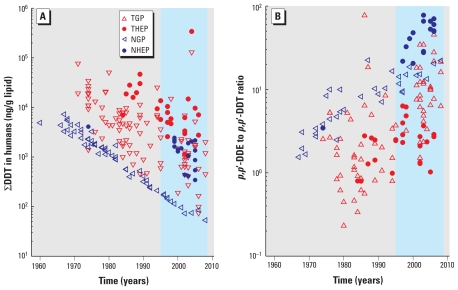
Temporal trends in human biomonitoring data. (*A*) Trends of ∑DDT. (*B*) Trends of the *p,p*′-DDE to *p,p*′-DDT ratio. Blue shaded area marks the time period investigated in the integrated exposure assessment (1995–2008). References for empirical data are given in the Supplemental Material, Table 7 (doi:10.1289/ehp.1002542).

Values of ∑DDT and of the *p,p*′-DDE to *p,p*′-DDT concentration ratio from the human biomonitoring data are shown in Figure 1A and Figure 1B, respectively. ∑DDT levels in the NGP show a declining trend with approximately first-order kinetics. Concentrations of ∑DDT in the TGP also show a declining trend but with a considerably larger variability compared with the NGP. ∑DDT concentrations of the THEP are consistently higher than concentrations in the TGP but also decline over time. Concentrations in the NHEP, which are based on measurements in various regions in Greenland, show higher variability than values for the NGP.

The *p,p*′-DDE to *p,p*′-DDT ratio increases with time for all populations except the THEP (Figure 1B). The *p,p*′-DDE to *p,p*′-DDT ratio differs significantly (ANOVA, *p* < 0.001) between the TGP and the THEP if only the data from 1995 to 2008 are considered; the median *p,p*′-DDE to *p,p*′-DDT ratios are 8.5 and 2.7 for the TGP and the THEP, respectively. The shaded area in Figure 1 corresponds to the time interval (1995–2008) considered in the geographically integrated exposure assessment.

### Geographically integrated exposure assessment

**Figure 2:**
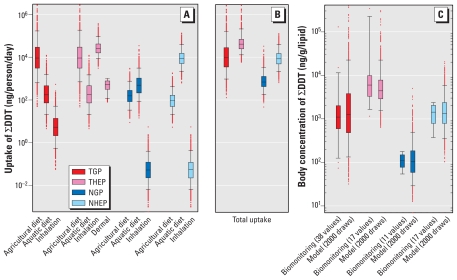
Results of the integrated exposure assessment representing the period of 1995–2008. Boxes represent median (central line) and 25th and 75th percentiles; whiskers mark 5th and 95th percentiles. (*A*) Daily uptake of ∑DDT from different exposure routes. (*B*) Total daily uptake resulting from combination of route-specific uptakes shown in (*A*). (*C*) Measured and modeled concentrations of ∑DDT in humans. Modeled distributions in (*C*) were calculated from total uptakes shown in (*B*). Because of efficient uptake of ∑DDT via the dietary and inhalation routes, our results, presented as uptakes, closely reflect intakes. References for empirical data are given in the Supplemental Material, Tables 1 and 7 (doi:10.1289/ehp.1002542).

Total uptake of ∑DDT is highest in the THEP, about equal in the TGP and NHEP, and lowest in the NGP (Figure 2B, Table 2). The total uptake in the THEP exceeds the total uptake in the TGP by about a factor of 5, and total uptake in the NHEP exceeds uptake in the NGP by a factor of 12.

The contributions of different exposure routes to total uptake of ∑DDT show important differences among the four populations (Figure 2A). In both Northern populations, dietary exposure via the aquatic food web dominates. This route represents the sum of exposure by consumption of marine and freshwater fish and, in the case of the NHEP, also includes marine mammals. In contrast, dietary exposure in the Tropics is mainly via the agricultural food web, which is the sum of exposure via dairy products, meat, and grains. Dietary uptake is the dominant exposure route for the TGP, but the dominant route for the THEP is inhalation: the median estimate of exposure by inhalation in the THEP is 2.5 times the estimated median exposure through diet. For all other populations, inhalation exposure is negligible.

Lipid-normalized concentrations measured in biomonitoring studies are shown in Figure 2C. Concentrations for 1995–2008 show highly significant (ANOVA, *p* < 0.001) differences between the TGP and THEP and the NGP and NHEP, respectively. Distributions of ∑DDT body concentrations derived from our estimated uptakes and the PK model are in very good relative and absolute agreement with these measured data: modeled and measured median concentrations differ by less than a factor of two, and the large differences between the general populations and the highly exposed populations are also indicated by the modeled data. Therefore, under the assumption that dietary uptake for the TGP and the THEP is similar (Sereda et al. 2009), inhalation exposure explains most of the significant concentration differences between the THEP and the TGP.

## Discussion

A key finding of our study is that exposure and levels calculated for the THEP are the highest among the four subpopulations examined and that they are due largely to uptake of ∑DDT via inhalation. In the median values shown in Figure 2B, inhalation accounts for 70% of the total uptake of the THEP. The remaining 30% is derived mostly from the agricultural diet. In the TGP, in contrast, the agricultural diet contributes 98% of total uptake. (These percentages of uptake refer to the median values. Note that there are situations where the aquatic diet dominates DDT uptake also in the TGP; see Figure 2A.) These findings are also consistent with observed *p,p*′-DDE to *p,p*′-DDT ratios, which are different for the TGP and the THEP: whereas the ratio changes drastically for the TGP in the recent period, the ratio has remained constantly low for the THEP (Figure 1B). A high ratio of *p,p*′-DDE to *p,p*′-DDT has been associated with a lack of exposure to freshly applied DDT (Sereda et al. 2009; Wong et al. 2005). This is because formulations of DDT contain approximately 75–85% *p,p*′-DDT [Agency for Toxic Substances and Disease Registry (ATSDR) 2002; Bouwman et al. 2006) and because metabolic conversion of *p,p*′-DDT to *p,p*′-DDE is slow in humans (Morgan and Roan 1971). Consequently, *p,p*′-DDE measured in the human body originates mainly from uptake of *p,p*′-DDE formed by degradation of *p,p*′-DDT in the environment (Morgan and Roan 1971; Kutz et al. 1976). In addition, intrinsic elimination in humans is about three times faster for *p,p*′-DDT than for *p,p*′-DDE (Ritter et al. 2009). Therefore, in the absence of exposure to fresh DDT, the fraction of *p,p*′-DDE increases with time because of differential uptake and differential elimination of *p,p*′-DDT and *p,p*′-DDE.

The relevance of nondietary exposure for individuals living in dwellings treated with IRS of DDT has also been pointed out by Sereda et al. (2009). Their conclusion was based on a comparison of biomonitoring data from two communities with the same food source, but one community living in IRS-treated dwellings and the other not. Our results corroborate this and indicate that under the assumption of similar dietary uptake for the TGP and the THEP, ∑DDT taken up via inhalation explains most of the difference in body concentrations between the TGP and the THEP.

Our estimate of uptake from inhalation is based on indoor air concentrations of ∑DDT (in this case consisting mainly of DDT) that were measured over a period of 240 days after a spraying event in both the gas and the particulate phase separately (Singh et al. 1992). These concentrations are on the order of 1–10 μg/m^3^, which is roughly 1,000 times higher than typical concentrations measured in tropical continental air and about 1 million times higher than typical concentrations measured in outdoor air in the North. These high concentrations are consistent with recent measurements in IRS-treated homes in South Africa (Bouwman et al. 2009). DDT concentrations in indoor air in the micrograms per cubic meter range were also obtained from a simple mass-balance model with DDT evaporating from treated walls as emission source [see Supplemental Material, Tables 1, 8, and 9 (doi:10.1289/ehp.1002542)].

No experimental studies that quantify the rate of uptake of DDTs from inhaled air in animals or humans are available (ATSDR 2002). We used uptake efficiencies of 100% for the gas phase and 44% for the particulate phase that were estimated by Volckens and Leith (2003) for *p,p*′-DDT. However, our results do not change markedly if the generic value of 75% for the inhalation uptake efficiency of both gas and particulate phase is used (European Commission 1996).

The low contribution of the aquatic diet to total uptake in the TGP and the THEP relative to the Northern populations is consistent with previous findings (Kannan et al. 1997). It is attributable to both lower average fish consumption [see Supplemental Material, Table 2 (doi:10.1289/ehp.1002542)] and lower bioaccumulation of DDTs in Tropical fishes (Kannan et al. 1995). However, in particular communities with high fish consumption and relatively high DDT concentration in fish, consumption of fish can also be an important source of DDT in Tropical regions (Barnhoorn et al. 2009; Kannan et al. 1992). Our results are based on point estimates of food consumption but also include situations with high contributions from the aquatic diet in tropical regions, as shown by the overlapping whiskers of the agricultural and aquatic diet in Figure 2A.

We also estimate the uptake from dermal exposure to contaminated soil and dust in IRS-treated dwellings (Equation 3). We find dermal exposure to be less important than uptake with food, but not negligible. The dermal uptake rate (Spalt et al. 2009), concentrations in indoor soil and dust (Herrera-Portugal et al. 2005), and the time spent indoors (Bouwman et al. 2009) are based on empirical data specific to DDT and IRS conditions. No empirical information specific to IRS was available for the surface area of skin exposed (AE) and for the soil and dust adherence (SDA). We assumed maximal and minimal AE values of 9,550 cm^2^ and 1,120 cm^2^, respectively. Maximum AE represents complete coverage of upper and lower extremities; minimal AE represents coverage of feet only. For the SDA we used 1 mg/cm^2^, which is about six times larger than hand loadings from soil-related activities such as gardening (U.S. Environmental Protection Agency 2009). Therefore, our assumptions concerning dermal exposure may be regarded as conservative.

### Model validation and sensitivity

Our assessment of total exposure is in good agreement with biomonitoring data from the same time period (Figure 2C).

Furthermore, our estimates of total uptake for the TGP, NGP, and NHEP, which closely reflect dietary intakes for these three populations, agree well with independent estimates from total diet studies from various regions (Table 2). No estimates of intake or uptake for the THEP were found in the literature.

Our assessment is based on average or median DDT concentrations from many individual studies [see Supplemental Material, Table 1 (doi:10.1289/ehp.1002542)]. These DDT concentrations were combined with point estimates of exposure factors to yield estimates of route-specific uptake (Figure 2A) and total uptake (Figure 2B). Accordingly, the ranges of uptake shown in Figure 2 do not represent the full variability of DDT uptake that may be found in the four populations. The full range of DDT uptake is wider than the ranges in Figure 2 because also exposure factors (food consumption rates, inhalation rates, times spent indoors) are variable and contribute to the variability of DDT uptake. Here it is important to note that the goal of our study is not to estimate the full ranges of DDT uptake. Our goal is to convert the variability in reported DDT concentrations in exposure media into ranges of uptake and body concentrations without introducing additional sources of variability, and then to compare the ranges of estimated body concentrations with DDT levels from biomonitoring data. Note that also the full range of DDT concentrations measured in humans in the four populations is wider than the ranges derived from the biomonitoring studies in Supplemental Material, Table 7 and shown in Figure 2C, because the biomonitoring studies included in Figure 2C report averages from groups that contained up to several hundred individuals (see Supplemental Material, Table 7). Variability within these groups is not shown in Figure 2C.

Although the ranges of DDT uptake that we determined here are not as wide as the true ranges, in the THEP and TGP there are situations where the acceptable daily intake (ADI) of 20 μg/kg/day (Bouwman et al. 2006), corresponding to 1.3 × 10^6^ ng/day for an adult of 65 kg, may be exceeded (Figure 2A). As a consequence of high uptake by adults, also infants may experience particularly high exposure via breast-feeding. Bouwman et al. (2006) calculated that the ADI for ∑DDT was exceeded up to 12.3 times for breast-fed children, emphasizing the need to consider infant health risks (Bouwman and Kylin 2009). However, as pointed out above, it is beyond the scope of this study to characterize specific cohorts of populations with particular exposure conditions, for example, children or fishermen with high fish consumption. Our results reflect the integration of average ∑DDT concentrations and food consumption data on large geographic scales.

Key parameters in the PK model are the elimination half-lives of *p,p*′-DDT and *p,p*′-DDE, but information about elimination of *p,p*′-DDT and *p,p*′-DDE is scarce (Ritter et al. 2009). Therefore, we took half-life estimates from Ritter et al. (2009), who used a broad empirical basis (long-term population data in Sweden) to derive elimination half-lives; furthermore, they employed a method that eliminates the confounding effect of ongoing exposure and yields intrinsic elimination half-lives, which are needed as input for PK models. Their half-life values agree within factors of 1.4 (*p,p*′-DDE) and 1.7 (*p,p*′-DDT) with values reported by other authors (Morgan and Roan 1972; Wolff et al. 2000).

Equations 1–3 and the PK model at steady state are multiplicative, except when different versions of Equation 2 are added to sum contributions from indoor and outdoor air. In these multiplicative relationships, doubling of any of the parameters while keeping the others constant will also double the output. For instance, changing the dose-weighted elimination half-life of ∑DDT from its lowest value (4.1 years) to the highest value (6 years) increases the modeled concentration by a factor of 6/4.1 = 1.46. However, measured median concentrations of the general population and the highly exposed population in the Tropics and the North differ by factors of 5.4 and 12, respectively (Figure 2C). Hence, the influence of this range of uncertainty in the elimination half-life is small compared with the concentration differences between the populations. In contrast, omitting the inhalation pathway in the THEP would cause a reduction in exposure by a factor of 3.5, shifting it to almost the same level as for the TGP.

### Time trends in biomonitoring data

We find that concentrations of ∑DDT decline from 1960 to 2008 for the TGP, THEP, and NGP (Figure 1A). Similar declining trends of ∑DDT in humans have previously been reported as integrated continental trends for Asia and the Middle East, Latin America, the United States and Canada, and Europe for the period 1960–1995 (Smith 1999). For the NHEP the trend is less clear. The biomonitoring data exhibit a large variability, reflecting differences in marine mammal consumption patterns among Inuit communities in Greenland (AMAP 2009). Trend assessments that were restricted to only one community show significant declines in concentrations of *p,p*′-DDE (AMAP 2009). However, these reductions in DDTs among Inuits in Greenland have been attributed mainly to decreasing consumption of traditional foods rather than to decreasing food contamination (Deutch et al. 2006). In addition, the decline of concentrations of many persistent chemicals, including DDTs, in fishes in the Great Lakes has recently been shown to be slowing (Carlson et al. 2010). Input from local and global sources likely contributes to this stabilization of contaminant levels. Sources of DDTs in the North may include continuing emissions from agricultural soils in North America (Bidleman et al. 2006) and ongoing long-range transport in air and oceans (Schenker et al. 2008). This indicates the need for a continued monitoring and assessment of concentration–time trends of legacy POPs in Northern regions.

## Conclusions

The need for strategies to further integrate and exploit the growing amount of monitoring data available for environmental pollutants has been identified as priority issue (Whylie et al. 2003). Our results show that integration of biomonitoring data on large geographic scales is possible. Median nonoccupational exposure to DDTs arising from IRS is about 60 times higher than current exposure of the general population living in countries such as Sweden or Canada. Inhalation of DDT is likely to be a dominant exposure route for people living in IRS-treated homes and needs to be considered in human health risk assessments. However, further research is needed to investigate other potential routes, including uptake via inhalation and ingestion of dust, dermal exposure via contaminated clothes, and transfer of DDT applied in IRS to food stored in the dwellings and subsequent consumption. Understanding nonoccupational exposure arising from IRS is crucial in developing exposure reduction strategies and mitigating health effects of DDT. In the North, median DDT uptake of indigenous populations with high consumption of marine mammals is 12 times higher than uptake of the general population. Trends in the North need to be assessed for slowdowns in the decline of concentrations, for changes in the DDE to DDT ratio, and for the effect of ongoing input of DDT used in the Tropics.

## Figures and Tables

**Table 1:** Exposure routes considered for the four populations.

			Inhalation		
Region	Population	Diet	Outdoor air	Indoor air	Dermal
Tropics	TGP	Yes	Yes	Yes^a^	No
	THEP	Yes	Yes	Yes	Yes
North	NGP	Yes	Yes	Yes^a^	No
	NHEP	Yes	Yes	Yes^a^	No

a Concentration in indoor air assumed to be equal to concentration in outdoor air.

**Table 2:** Comparison of total uptakes^a^ (nanograms per person per day) from this study with results from total diet studies.

Study details	Year	TGP	THEP	NGP	NHEP	Reference
Sweden (market based)	1999			523		Darnerud et al. 2006
Canada (total diet)	1998			490		Rawn et al. 2004
Sweden (nonvegetarian diet)	1990			2,240		Vaz 1995
Greenland	2004				10,120	Deutch et al. 2006
Vietnam	1990–1991	19,000				Kannan et al. 1992
India (vegetarian diet)	2001	2,200				Battu et al. 2005
India (nonvegetarian diet)	2001	13,600				Battu et al. 2005
India (vegetarian diet)	2002	8,170				Battu et al. 2005
India (nonvegetarian diet)	2002	27,200				Battu et al. 2005
Integrated assessment (P25)	1995–2008	3,200	27,500	425	5,040	This study
Integrated assessment (P50)	1995–2008	9,580	43,500	722	9,360	This study
Integrated assessment (P75)	1995–2008	29,300	75,900	1,290	16,600	This study

P25, P50, P75 are the 25th, 50th, and 75th percentile of the uptake distribution.

a Because uptake efficiency of DDT in the gastrointestinal tract is high (0.9), estimates of uptake and intake differ only by 10% for the dietary route, which dominates exposure of the TGP, NGP, and the NHEP; therefore, consistency is assured.
